# Prevalence of IgG antibodies against Malawi polyomavirus in patients with autoimmune diseases and lymphoproliferative disorders subjected to bone marrow transplantation

**DOI:** 10.3389/fimmu.2023.1293313

**Published:** 2024-01-17

**Authors:** Jérôme T. J. Nicol, Elisa Mazzoni, Maria Rosa Iaquinta, Raffaella De Pace, Pauline Gaboriaud, Natalia Maximova, Carolina Cason, Eleonora De Martino, Chiara Mazziotta, Pierre Coursaget, Antoine Touzé, Valentina Boz, Manola Comar, Mauro Tognon, Fernanda Martini

**Affiliations:** ^1^ UMR 1282 ISP Team Biologie des Infections à Polyomavirus, Faculty of Pharmacy, University of Tours, Tours, France; ^2^ Department of Chemical, Pharmaceutical and Agricultural Sciences, University of Ferrara, Ferrara, Italy; ^3^ Department of Medical Sciences, University of Ferrara, Ferrara, Italy; ^4^ Onco-Hematology Division, Institute for Maternal and Child Health, IRCCS “Burlo Garofolo”, Trieste, Italy; ^5^ Department of Advanced Translational Microbiology, Institute for Maternal and Child Health, IRCCS “Burlo Garofolo”, Trieste, Italy; ^6^ Laboratory of Pediatric Immunology, Institute for Maternal and Child Health, IRCCS “Burlo Garofolo”, Trieste, Italy; ^7^ Department of Pediatrics, Institute for Maternal and Child Health, IRCCS “Burlo Garofolo”, Trieste, Italy; ^8^ Department of Medical Sciences, University of Trieste, Trieste, Italy; ^9^ Laboratory for Technologies of Advanced Therapies, University of Ferrara, Ferrara, Italy

**Keywords:** MWPyV, antibody, autoimmune diseases, lymphoproliferative disorders, prevalence

## Abstract

**Introduction:**

Human polyomaviruses (HPyVs) cause persistent/latent infections in a large fraction of the population. HPyV infections may cause severe diseases in immunocompromised patients. Malawi polyomavirus (MWPyV) is the 10th discovered human polyomavirus (HPyV 10). MWPyV was found in stool samples of healthy children. So far, the few investigations carried out on HPyV 10 did not find an association with human disease.

**Methods:**

In this study, to verify the putative association between MWPyV and human diseases, MWPyV seroprevalence was investigated in patients affected by i) lymphoproliferative disorders (LPDs) and ii) immune system disorders, i.e., autoimmune diseases (ADs), and in iii) healthy subjects. An indirect ELISA, employing virus-like particles (VLPs) to detect serum IgG antibodies against MWPyV/HPyV 10, was carried out. The study also revealed the prevalence of another polyomavirus, Merkel cell polyomavirus (MCPyV).

**Results:**

Sera from patients with distinct autoimmune diseases (*n* = 44; mean age 20 years) had a prevalence of MWPyV antibodies of 68%, while in patients with lymphoproliferative disorders (*n* = 15; mean age 14 years), subjected to bone marrow transplantation, the prevalence was 47%. In healthy subjects (*n* = 66; mean age 13 years), the prevalence of MWPyV antibodies was 67%. Our immunological investigation indicates that MWPyV/HPyV 10 seroconversion occurs early in life and MWPyV/HPyV 10 appears to be another polyomavirus ubiquitous in the human population. A significantly lower MWPyV antibody reactivity together with a lower immunological profile was detected in the sera of LPD patients compared with HS2 (*p < 0.05) (Fisher’s exact test). LPD and AD patients have a similar MCPyV seroprevalence compared with healthy subjects.

**Discussion:**

MWPyV seroprevalence indicates that this HPyV is not associated with lymphoproliferative and autoimmune diseases. However, the ability to produce high levels of antibodies against MWPyV appears to be impaired in patients with lymphoproliferative disorders. Immunological investigations indicate that MWPyV seroconversion occurs early in life. MCPyV appears to be a ubiquitous polyomavirus, like other HPyVs, in the human population.

## 1 Introduction

The human polyomaviruses (HPyVs) belong to the *Polyomaviridae* family. They are small viruses (45 nm), with a double-stranded DNA genome, which are widespread in humans reaching high prevalence, varying between 60% and 100% in the worldwide adult healthy populations (1). So far, from 1971 to 2019, 15 different polyomaviruses have been identified in humans (2). Polyomaviruses represent part of the normal skin microflora of healthy individuals. However, HPyVs may cause diseases of different severity in patients/subjects with i) impaired immune functions due to organ transplant, ii) HIV/AIDS, iii) age, or iv) cancer (3).

Indeed, epidemiological studies reported associations of JCPyV, BKPyV, MCPyV, and TSPyV with malignancies of different histotypes. In addition, molecular biology studies showed viral integration and/or large T antigen (TAg) expression in tumor samples. Therefore, the International Agency for Research on Cancer (IARC) classified these four HPyVs as possible human carcinogens (4). Among pathogenetic human polyomaviruses, JC polyomavirus (JCPyV) may cause demyelinating disease known as progressive multifocal leukoencephalopathy (PML) (5). Merkel cell polyomavirus (MCPyV) is associated with Merkel cell carcinoma (MCC), a highly aggressive skin cancer (6). BK polyomavirus (BKPyV) induces nephropathy in kidney transplant recipients (KTRs) (7). A recent study explored the clinical profile, outcomes, and follow-up experiences of KTR patients who developed BKPyV after COVID‐19 (8). The authors suggest screening for BKPyV in all renal transplant patients with active COVID-19 infection as a safe option to avoid complications because BKPyV causes complex changes in immunity and weakens the immune response, which could potentially aggravate the immune/graft injury often present during COVID-19 infection (8). It has been reported that HPyVs may infect asymptomatic subjects during early childhood, followed by a latency stage. In a peculiar condition of the host, such as immunosuppressive status, HPyVs may reactivate (3, 9, 10). HPyV6, HPyV7, and TSPyV have been associated with rare skin lesions in immunosuppressed patients (11). However, HPyV9, HPyV10/MWPyV, and Saint Louis polyomavirus (STLPyV) have not been convincingly associated with any disease (4).

Among new HPyVs, little attention has been dedicated to the HPyV10/MWPyV (4). MWPyV seems to be ubiquitous in human populations (12), while its pathogenic role if any remains to be investigated. The MWPyV genome is composed of 4,927 bp (13). The complete DNA sequencing showed that the MWPyV genome belongs to the genus *Delta* of the *Polyomaviridae* family. Its genome can be divided into three regions: the regulatory region, the early region, and the late region (13). The early region encodes the large T antigen (LTAg) and small T antigen (STAg), while the late region is expressed after viral replication and encodes the structural proteins of the capsid VP1, VP2, and VP3 (13, 14). The MWPyV small T antigen (ST) binds protein phosphatase 2A (PP2A), and the large T antigen (LT) binds pRb, p107, p130, and p53. However, MWPyV LT and ST antigens did not enhance the cellular proliferation, compared with th control (15).

MWPyV was initially identified by shotgun pyrosequencing of DNA extracted from viral particles isolated from a stool sample collected from a 1-year-old healthy child in Malawi (13). Another study, using an unbiased deep sequencing approach, identified MWPyV in stool samples from children affected by acute diarrhea (16). In addition, MWPyV has been detected in fecal material from Brazilian children, suggesting the fecal–oral transmission route for this virus (17). A prevalence of 18.4% has been reported for HPyV10 DNA detected in benign and malignant tonsil biopsies while absent in normal tissues (18). In immunocompromised patients, MWPyV can result in a subclinical infection in children, with persistent or intermittent shedding (19). MWPyV was reported to be present in the skin (20), but melanoma samples tested HPyV10-negative (21). The skin appears transiently infected, and it does not appear to be a permanent HPyV10 reservoir (4). HPyV10 was isolated from condylomas on the buttocks of patients suffering from a rare genetic disorder, known as warts, hypogammaglobulinemia, infections, and myelokathexis (WHIM) syndrome (22). MWPyV seroconversion occurs early in life, most commonly in children 1–2 years old, as observed for other HPyVs (12, 20). A seroepidemiological survey carried out on a large sample size in samples of individuals from 1 to ≥80 years old documented MWPyV circulation with a trend of frequency similar to JCPyV (5, 23) and BKPyV (24, 25). In our previous study, a MWPyV seroprevalence of 41.8% was revealed in an adult population (12). Berrios et al. (15) investigated the prevalence of serum immunoglobulin G (IgG) antibodies against MWPyV/HPyV10 in a cohort of 500 subjects in Denver, CO, USA. Their immunologic data indicate a higher prevalence of HPyV10 antibodies in Colorado compared with healthy subjects from Italy (12, 15). These differences may reflect the distinct technical approaches employed in the two studies or the two different populations studied, but the results did not differ substantially from each other.

Lymphotropic viruses, including the Epstein–Barr virus (EBV) and human herpesvirus-8 (HHV8), have been associated with the pathogenesis of distinct immunodeficiency-associated lymphoproliferative disorders (LPDs) (26). LPD is a specific category proposed in 2016, as a revised WHO classification concerning lymphoid neoplasms (27). Several factors, including autoimmune disease (AID) activity, immunosuppressive drugs (ISD) usage, and aging, influence the development of other iatrogenic immunodeficiency-associated-LPD (OIIA-LPD), resulting in complicated clinical courses and outcomes (28). Immunosuppressed patients may be affected by a wide range of LPD, from self-limiting disorders to malignant lymphoid proliferations (26). These LPDs may be associated with systemic immune disorders, develop following organ transplantation, or occur in the background of other forms of iatrogenic immunosuppression (27).

Given the potentially serious implications of polyomavirus infections in immunocompromised populations and the possible role of these viruses in cell transformation, it is important to further understand the prevalence of MWPyV. Since HPyV can be transmitted by blood transfusion in the immune-compromised host, herein, we evaluated the seroprevalence of antibodies against MWPyV in patients affected by different LPD/BMT and AD. The main goal of this investigation was to verify whether an association exists between MWPyV infection and LPD/BMT and AD diseases. To evaluate the effect of an immunosuppressive regimen on LPD patients, the status of sera IgG against MWPyV was evaluated by enzyme-linked immunosorbent assay (ELISA) using virus-like particles (VLPs) as antigen (12, 14).

## 2 Materials and methods

### 2.1 Samples and clinical data

Samples reported before (29) were from our archive (**Table 1**). As reported in an earlier investigation, serum samples from patients (*n* = 59) were from the Pediatric Department of the Children Hospital “Burlo Garofolo,” Trieste, Italy. Written informed consent was obtained from the patients/individuals if >18 years old or from the parents when <18 years old. Patients (*n* = 59) included in this study are i) children and adolescents/young adults affected by lymphoproliferative disorders, subjected to bone marrow transplant (LPD/BMT, *n* = 15; mean age =14, 8–19 years old); and ii) children, adolescents, and adults with different autoimmune diseases (ADs, *n* = 44; 7–43 years old).

**Table 1:** Seroprevalence of Malawi polyomavirus (MWPyV) in patients and healthy subjects.

Serum sample	Number of patients/subjects	Mean (range), years	Male %	Number of positive samples (%)	*High antibodies positive High positive sera/sera positive
Patients	59	18 (7–43)	45	37 (63)	7/37 (19)
AD	44	20 (7–43)	39	30 (68)	7/30 (23)
HS1	31	17 (11–33)	23	21 (68)	7/21 (33)
LPD/BMT	15	14 (8–19)	67	7 (47)	0/7 (–)*
HS2	66	13 (6–33)	43	44 (67)	18/44 (40)

The prevalence of Malawi polyomavirus antibodies in patients was not statistically significant (p > 0.05).

AD, autoimmune disease; LPD, lymphoproliferative disorder; BMT, bone marrow transplantation; HS, healthy subjects.

*High levels of antibodies were observed when the optical density (OD) values of the sera exceeded the third quartile of OD values (0.638 OD), the value used as the cutoff for high levels of antibodies. The prevalence of patients with high levels of Malawi polyomavirus antibodies in AD patients was not statistically significant compared with HS1 (p > 0.05), while the prevalence of patients with high levels of Malawi polyomavirus antibodies in LPD patients was statistically significant compared with HS2 (*p < 0.05) (Fisher’s exact test).

In order to investigate the MWPyV seroprevalence in the two groups of patients, with a different mean age, two control groups were selected (HS1, HS2) with distinct mean ages. This selection allowed us to match the different mean ages of the two groups of patients, AD (mean age 20 years old) and LPD (mean age 14 years old), with those of the control, the HS1 cohort (*n* = 31; mean age of 17 years old, range 11–33 years old), and HS2 (*n* = 66; mean age of 13 years old, range 6–33 years old), respectively.

AD patients have a median age of 20 years old; LPD patients have a median age of 15 years old; healthy subjects have a median age of 17 years (HS1) and 10 years old (HS2), respectively. The different cohorts analyzed by unpaired *t*-test and Mann–Whitney test show no statistical differences between the mean age of AD-affected patients and HS1 and between LPD-affected patients and HS2 (*p* > 0.05). The mean age is reported in **Table 1**.

The healthy subjects (HS) were analyzed for a routine check-up analysis. The hospital records indicated that they were in good health at the time of blood collection. Sera were taken from discarded laboratory specimens after routine analyses before the incineration. Anonymously collected sera were coded indicating the age and gender only.

The case group consisted of 59 patients, consisting of 44 patients affected with ADs treated with a monthly infusion of biological drugs, including infliximab or intercept as monotherapy, without experience of neurological deficits or brain lesions, together with 15 patients affected by LPD, subjected to BMT, coded as LPD/BMT.

The transplant conditioning was performed with nelarabin, total body irradiation (TBI) 12 Gy in 6 doses, thiotepa (ThT), cyclophosphamide (Cy), and rabbit antithymocyte globulin (ATG), followed by infusion of 5.1 × 10^8^ total nuclear cells (TNCs)/kg from a matched unrelated donor. Graft versus host disease prophylaxis was done with cyclosporine (Cs-A) and mycophenolate mofetil (MMF), while antiviral prophylaxis was done with acyclovir 30 mg/kg/day i.v. All LPD/BMT patients had a history of blood transfusions before the transplantation.

Similarly, patients with different ADs were not affected by other diseases. The sera obtained from AD patients include chronic glomerulonephritis (*n* = 1), chronic inflammatory diseases (*n* = 1), scleroderma cutaneous (*n* = 1), liver transplantation (tyrosinemia type 1) (*n* = 1), juvenile idiopathic arthritis (JIA) (*n* = 18), ulcerative rectocolitis (*n* = 3), Crohn’s disease (*n* = 16), Behcet’s disease (*n* = 1), pars planitis (*n* = 1), and liver transplantation in hemochromatosis (*n* = 1). Serum samples (*n* = 15) from children, adolescents, and young adults affected by different lymphoproliferative disorders were affected by acute lymphoblastic leukemia (ALL) (*n* = 4), juvenile myelomonocytic leukemia (JMML) (*n* = 5), acute myeloid leukemia (*n* = 5), and severe aplastic anemia (*n* = 1). The demographic and clinical characteristics of the patients’ cohort are summarized in **Tables 1**, **2**. The study cohorts, from the same area (northeastern Italy), were analyzed using ELISA.

**Table 2:** The MWPyV antibody-positive samples detected in lymphoproliferative disorder/bone marrow transplant and autoimmune disease patients.

Diagnosis	MWPyV antibody-positive samples/samples analyzed (%)
**All patients** Male Female	**37/59 (63)** 15/27 (55) 22/32 (69)
**Different lymphoproliferative disorder/bone marrow transplant patients**	**7/15 (47)**
Acute lymphoblastic leukemia (ALL)	4/4 (100)
Juvenile myelomonocytic leukemia (JMML)	2/5 (40)
Acute myeloid leukemia (AML)	1/5 (20)
Severe aplastic anemia	0/1
**Autoimmune disease patients**	**30/44 (68)**
Chronic glomerulonephritis	1/1 (100)
Chronic inflammatory diseases	1/1 (100)
Scleroderma cutaneous	1/1 (100)
Liver transplantation (tyrosinemia type 1)	1/1 (100)
Juvenile idiopathic arthritis (JIA)	14/18 (78)
Ulcerative rectocolitis	2/3 (67)
Crohn’s disease	10/16 (62)
Behcet’s disease	0/1
Pars planitis	0/1
Liver transplantation in hemochromatosis	0/1

### 2.2 MWPyV VP1 ELISA

In order to detect the IgG antibodies against the MWPyV, the sera from AD and LPD/BMT patients and healthy subjects, for a total of 156 samples, were investigated using a VLP-based ELISA previously developed and validated (12, 14). Serum samples were analyzed as described before (12). The MWPyV VP1 coding sequence (GenBank accession no. NC_018102.1) was codon-optimized for expression in *Spodoptera frugiperda* cells (GenScript, Piscataway, NJ, USA) and used to generate a recombinant baculovirus. Purified MWPyV VLPs [100 ng/well in phosphate-buffered saline (PBS)] were used to sensitize microplates (MaxiSorp; Nunc) overnight at 4°C. Briefly, sera were diluted 1:100, and peroxidase-conjugated anti-human IgG (Southern Biotech, CliniSciences, Nanterre, France) diluted 1:20,000 was used to detect human IgG binding (12). In a previous study by the same groups, a correlation analysis was carried out (12). Data suggested that no cross-reactivity occurs between MWPyV and the five human polyomaviruses tested. In addition, a cutoff value of 0.199 was established, and this value was obtained by plotting the net optical density (OD) value of 116 subjects with age under 10 years old. As previously reported (12), a histogram of the ELISA OD values in 1- to 10-year-old children (data not shown) revealed a bimodal distribution of seroreactivity. The cutoff point for MWPyV positivity was therefore set at 0.199 (mean of the lowest distribution of OD values plus 2 standard deviation).

### 2.3 Serum MCPyV antibody levels

The indirect ELISA was developed and validated to detect specific IgGs against MCPyV. MCPyV VP1 S and VP2 F peptides/mimotopes were employed (Thermo, Milan, Italy) (6, 30). Plates were coated with 5 μg of peptide for each well and diluted in 100 μl of Coating Buffer 1X (Candor Bioscience, Wangen, Germany). Each well was coated with 5 μg of one specific peptide, either S or F. Plates were left at 4°C for 16 h and then rinsed three times with washing buffer (Candor Bioscience, Wangen, Germany). The blocking phase was performed using 200 μl/well of blocking solution (Candor Bioscience, Wangen, Germany) at 37°C for 90 min. Wells were rinsed three times. Each well was covered with 100 μl containing 1:20 diluted sera in a low cross-buffer (Candor Bioscience, Wangen, Germany). Plates were rinsed three times before adding secondary Ab. Plates were incubated at room temperature (RT) for 90 min with a goat anti-human IgG heavy (H) and light (L) chain-specific peroxidase conjugate (Calbiochem-Merck, Germany). Wells were rinsed three times, and then 100 μl of 2,2′-azino-bis(3-ethylbenzothiazoline-6-sulfonic acid) (ABTS) solution (Sigma-Aldrich, Italy) was added to each well. After 45 min at RT, plates were read by a spectrophotometer (Thermo Electron Corp., Finland) at a wavelength of 405 nm. The color intensity in the wells was determined by OD reading. The cutoff of each peptide was determined in each ELISA run, as the mean of the OD readings of *n* = 3 negative control sera, adding three SDs of the mean (mean + 3 SDs), as described. Sera were considered MCPyV-positive when reacting to both S and F peptides. Control sera in each plate were included as reported (6, 30).

### 2.4 Total IgG values

Total IgG concentrations in the serum samples of LPD patients (*n* = 15) and AD patients (*n* = 17) were assessed using the commercial kit “Human IgG ELISA Kit” according to the manufacturer’s instructions (catalog number RAB0001; Millipore, Milan, Italy) (7, 31). The serum samples analyzed for total sera IgG variability were chosen from all samples, in an equal number of sera for the two different cohorts analyzed (LPD and AD) in order to be representative of the individual cohort. In addition, the samples within the same court were chosen randomly. The ELISA plate was read spectrophotometrically (Thermo Electron Corp., model Multiskan EX, Finland) at a wavelength of 450 nm. The lower threshold for detection of IgG with this method is 20 pg/ml. The reference intervals for healthy adults were IgG 700–1,600 mg/dl (32, 33).

### 2.5 Statistical analysis

The prevalence of MWPyV-positive serum samples from patients affected by LPD/BMT and AD was compared with the prevalence detected in healthy individuals. Moreover, to investigate variations in antibody levels according to gender and pathology, samples were considered as having high levels of antibodies when the OD value was greater than that of the third quartile of seropositive samples (OD = 0.683). The prevalence of HPyV10-positive serum samples with high levels of antibodies from patients affected by LPD/BMT and AD was compared with the prevalence detected in healthy individuals. To determine the significance between the two groups, Fisher’s exact test was used. The serologic profile of serum antibody reactivity to MWPyV was statistically analyzed with ANOVA multiple comparisons test. For all tests, *p*-value was considered to be statistically significant when less than *p <*0.05. Unpaired *t*-test and Mann–Whitney test were used to compare the mean age between AD patients and HS1 and between LPD and HS2 (*p* > 0.05). All computational analyses were performed with Prism 10.0 (GraphPad software).

## 3 Results

### 3.1 Malawi polyomavirus seroprevalence in AD- and LPD-affected patients

In this study, we investigated the prevalence of IgG antibodies against Malawi polyomavirus in children/young adults affected by autoimmune diseases and in patients affected by different LPDs, including leukemia, subjected to BMT. The cohort of the negative control was represented by healthy children/young adults, with the same mean age of the two cohorts of patients under analysis. The prevalence of serum antibodies against MWPyV investigated in two cohorts of healthy subjects was 68% in HS1 (*n* = 21/31) and 67% (*n* = 44/66) in HS2 (**Table 1**). The prevalence of serum antibodies against MWPyV investigated in all patients (LPD/AD) was 63% in HS1 (*n* = 37/59) (**Table 1**). In LPD/BMT patients, the overall prevalence of IgG antibodies against this polyomavirus was 47% (*n* = 7/15). The different prevalence rates were not significant between LPD/BMT versus the cohort of HS2 with similar age (*p* > 0.05) (**Table 1**). In the cohort of AD patients, serum antibodies against MWPyV were identified with an overall prevalence of 68% (*n* = 30/44, 68%) (**Table 1**). Moreover, it should be noted that the prevalence of IgG antibodies against MWPyV was similar in patients affected by autoimmune diseases (68%) versus the cohort of HS1 (*n* = 21/31, 68%), the control group (*p* > 0.05).

### 3.2 Malawi polyomavirus reactivity levels

In order to investigate the variations in antibody level according to age or pathology, as observed in previous reports (12, 14), samples were considered as having high levels of antibodies when the OD value was greater than that for the third quartile of seropositive samples (OD > 0.638). Age- and gender-adjusted odds ratio estimates (OR*) with 95% confidence intervals were calculated to assess the association between high reactivity, gender, and AD pathology (**Table 1**). High reactivity (OD > 0.638) was not associated with gender (*p* > 0.99) (**Table 1**). The prevalence of the high titer of IgG antibodies was 33% (*n* = 7/21) in the sera of HS1 and 40% (*n* = 18/44) in HS2. The prevalence of AD sera with a high titer of IgG antibodies was 23% (*n* = 7/30), while none of the sera from LPD/BMT patients had a high titer of IgG antibodies (**Table 1**). The overall data show that high reactivity against Malawi polyomavirus, reported as IgG antibody level, was not associated with AD patients (*p* > 0.05), while the prevalence of patients with high antibodies positive against Malawi polyomavirus in LPD patients was statistically significant compared with HS2 (*p < 0.05) (Fisher’s exact test).

**Figure 1:**
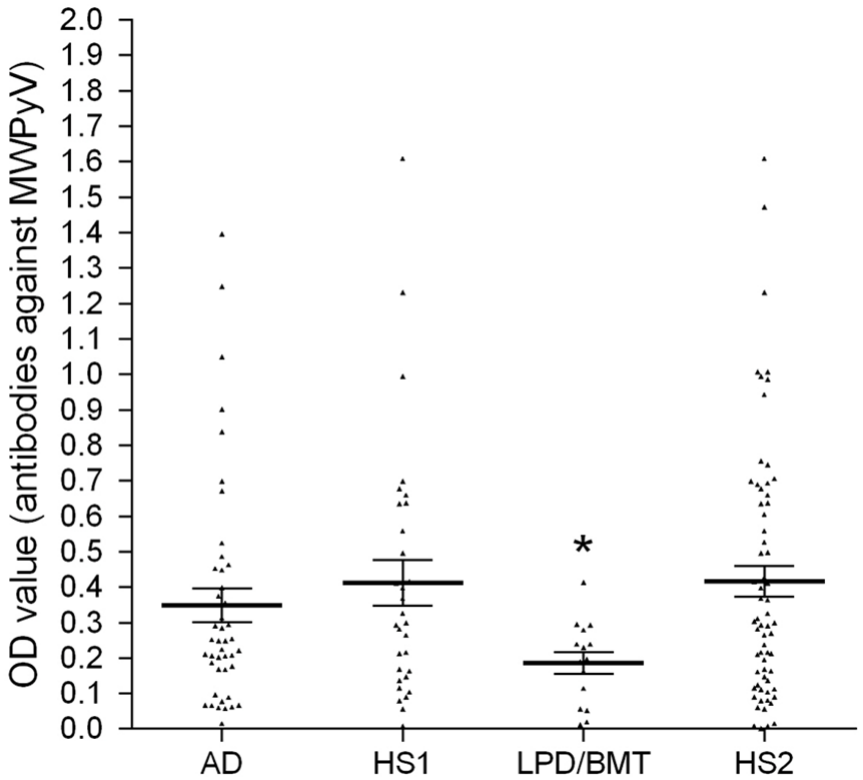
Serological profile of serum antibody reactivity to MWPyV VLPs. The immunological data were obtained by analyzing the serum samples of LPD/BMT patients, AD patients, and HS1/HS2, employed as control. In the scatter dot plotting, each plot represents the dispersion of OD values to a mean level indicated by the line inside the scatter with the SEM for each group of patients/subjects analyzed. The statistical analysis was performed with an analysis of variance with ANOVA and Dunnett’s multiple comparisons test. The mean OD in the sera from LPD/BMT patients was lower than the mean OD of sera from the HS groups, the control (*p < 0.05).

The MWPyV antibody-positive samples in patients affected by different lymphoproliferative disorders/bone marrow transplantation and autoimmune diseases are reported in **Table 2**. The antibody analysis shows that no pathological subtype in the LPD and/or AD cohorts is associated with an HPVyV 10 infection. The proportion of female (69%) and male (55%) patients, who were immune-responsive to the MWPyV infection, is similar. The determination of the antibody levels was evaluated as a serological profile of the optical densities (ODs) measured in ELISA. The antibody levels reported as ODs measured in the different cohorts showed that the serological profile of LPD/BMT sera is significantly lower than that of AD patients and healthy subjects (HS2). The serologic profile of LPD/BMT differs statistically from the serologic profile of healthy subjects (*p* < 0.05) (**Figure 1**). No difference was revealed between the serologic profile of AD and HS1 (**Figure 1**). The lower prevalence of IgG antibodies and the lower immunologic profile in subjects with a higher level of antibodies against MWPyV, detected in LPD/BMT patients, compared with young HS, could be due to the disease or the effect exerted by the different therapies used for bone marrow transplantation management.

### 3.3 Serum anti-MCPyV IgG levels

The sera from LPD and AD were tested by indirect ELISAs for IgG reactive to MCPyV. In the patient cohorts, the prevalence of seropositivity for both S/F peptides was 47% (7/15) in LPD and 49% (20/41) in AD patients. A similar seroprevalence was found in the HS cohort compared with patient cohorts as previously reported (*p* > 0.05) (6). Indeed, in previous studies, the prevalence of anti-MCPyV IgGs tested in HS, aged 11–15 years old, was 60.5%, and in HS, aged 18–30 years old, the prevalence was 63.1% (6, 30).

### 3.4 Total serum IgG

Total IgG concentrations in the serum samples of LPD patients (*n* = 15) and AD patients (*n* = 17) were assessed using the commercial kit “Human IgG ELISA Kit” according to the manufacturer’s instructions. The IgG levels (mean value ± SEM; mg/dl) present in the serum samples are i) 401 ± 236 mg/dl (LPD patients) and ii) 245 ± 155 mg/dl (AD patients). As reported before, total IgG values detected in the patient cohorts were lower compared with HS (7, 32, 33).

## 4 Discussion

HPyVs are a growing challenge in immunocompromised patients given the increasing number of HPyVs (to date, *n* = 15) and their pathogenic potential. In this investigation, MWPyV seroprevalence was determined in patients/individuals. The approach employed in this study was similar to that used in a previous epidemiological investigation with 825 sera from normal subjects, aged 1 to 100 years old (12, 14).

In this investigation, healthy subjects with a mean age of 17 and 13 years had a seroprevalence against MWPyV of 68% and 67%, respectively. In this study, MWPyV infection appears relatively common, since a seroprevalence of 67%–68% was observed in HS2 (mean age 13 years old) and HS1 (mean age 17 years old). No statistically significant difference in MWPyV seroprevalence was detected in i) the cohorts of patients/individuals and ii) gender (4). Studies carried out in different human populations indicated that HpyV infections are common in healthy individuals. Indeed, seroprevalence data indicate wide ranges from 23% of HpyV12 up to 90% for BKPyV, JCPyV, KIPyV, WUPyV, and TSPyV (4). Our previous study reported the seroepidemiology of Malawi polyomavirus. The investigation was carried out using a VLP-based ELISA (12). MWPyV seroprevalence was determined in 825 subjects from 1 to 100 years old. These data suggest that i) MWPyV infection occurs early in life and (ii) MWPyV seroprevalence reaches 42% in adulthood. The seroprevalence of healthy subjects did not differ substantially from that (58%) reported before for MWPyV detected in children/adolescents 10–14 years old (12, 14). The MWPyV serum antibody prevalence, reported herein, indicates that MWPyV infection occurs early in life, as observed for other human polyomaviruses (12). In a previous investigation (34), we suggested that HpyV may occur by vertical transmission. Indeed, BKPyV, JCPyV, and SV40 DNA sequences and IgG antibodies were detected in samples from female patients and their offspring, suggesting a potential risk of diseases in newborns. Further studies are required to elucidate the transmission mode of MWPyV in humans and its potential involvement in human diseases, including cancers, in both immunocompetent and immunocompromised patients.

In this study, we show that the overall MWPyV seroprevalence in patients affected by lymphoproliferative disease-bone marrow transplantation and autoimmune disease is 63%. Specifically, the MWPyV antibodies detected in LPD and AD patients were 47% and 68%, respectively. Patient cohorts affected by neoplastic and autoimmune diseases have no seroprevalence differences between them and healthy subjects with similar age (HS1, HS2).

The seroprevalence revealed in LPD patients is similar (47%) to that observed in healthy adults. The seroprevalence revealed in AD patients, with a mean age of 20 years (68%), is higher than that reported before for another cohort (12) and in this study for HS of the same age. Our immunological study indicates that there is no association between HpyV10 infection and LPD-BMT. In this context, it should be recalled that Herberhold et al. (18) reported a statistically significant difference between the prevalence of HpyV10 DNA in malignant and non-malignant tissues.

In order to investigate the variations in antibody levels, according to pathologies observed for other polyomaviruses, samples were considered as having high levels of antibodies when the OD value was greater than that for the third quartile of seropositive samples (OD = 0.628). Odds ratio estimates (OR*) with 95% confidence intervals were calculated to assess the association between high reactivity, gender, and pathology. Nicol et al. reported that the third quartile of OD values of MWPyV-seropositive samples, used as a cutoff for high antibody levels and high reactivity, was negatively associated with age. Interestingly, none of the sera from LPD patients exceeded the cutoff imposed to discriminate high reactivity (12). The prevalence of patients with higher levels of Malawi polyomavirus antibodies in AD patients was not statistically significant compared with HS1, while the prevalence of patients with higher levels of Malawi polyomavirus antibodies in LPD patients was statistically significant compared with HS2.

The serological profile of serum antibody reactivity to MWPyV of LPD/BMT patients was statistically lower compared with HS1. In agreement with this observation, the serological profile of ODs observed in the LPD cohort is statistically lower than in the AD cohort and healthy subjects. The immunosuppressive therapies used for BMT management could be responsible for the lower anti-MWPyV serum antibodies. In this regard, it has been demonstrated that pharmacological immunosuppressive therapy could modify the immune response to another polyomavirus, such as MCPyV (35), increasing the risk of MCC onset.

LPD patients present a lower level of total IgG in their sera, compared with healthy subjects of the same age, as previously reported (7). Leukemia patients are immunocompromised due to the disease state, which involves clonal expansion of abnormal lymphoid progenitors that are undifferentiated with abnormal functions (36). The most common and clinically relevant impact of CLL on the immunological status of affected patients is hypogammaglobulinemia (37). The reduction of immunoglobulins is present in up to a third of people with CLL at diagnosis and a further third develop hypogammaglobulinemia as the disease progresses or due to the administered therapy (37). Current guidelines recommend immunoglobulin replacement therapy (IgRT) to reduce the risk of bacterial infections and hospitalization in patients with recurrent severe infections and low levels of IgG. We detected a reduction in IgG levels in the sera of AD patients, too. In our experience, the total IgG values detected in patients with distinct diseases, including patients affected by multiple sclerosis (MS) and other inflammatory neurologic disorders (OINDs), did not differ statistically from healthy subjects.

To date, there are few data on the association between HPyV10 and human diseases. Although this HPyV has been detected in stool samples from children, there are no data supporting its association with pediatric gastroenteritis. At the same time, HPyV10 excretion in feces indicates that the fecal–oral transmission is one of the routes of MWPyV infection in humans (17).

Leukemia patients are more susceptible to a wide range of infections. The main causes of immunodeficiency are known to be aging, human immunodeficiency virus infection, and treatment with immunosuppressant drugs used for autoimmune diseases and after organ transplantation. There is a high frequency of viremia by a single virus and viremia by multiple viruses at the time of diagnosis of acute lymphoblastic leukemia in pediatric patients (38). Viral infections and reactivations are common and may result in severe complications (38). In this study, no infections with other polyomaviruses, such as MCPyV, in LPD patients were revealed. In our study, LPD and AD patients have a similar MCPyV seroprevalence compared with healthy subjects, as previously reported.

One limitation of this pilot study is the small sample size. Additional investigations are required with larger cohorts of patients/subjects to clarify whether MWPyV is associated with lymphoproliferative disorders, such as LPD/BMT or autoimmune diseases, as well as other human diseases.

To date, there are few data on the association between HPyV10 and human diseases. Our immunological investigations indicate specific dysregulations in IgG Abs against MWPyV in sera from LPD/BMT-affected patients. The serologic profile of serum antibody reactivity shows that the antibody titer against MWPyV in LPD/BMT sera is lower than in healthy subjects. This result is in agreement with the absence of high levels of Malawi polyomavirus antibodies in LPD patients. We may speculate that the low MWPyV seroprevalence detected in LPD/BMT patients could be due to an impaired immune response against this human polyomavirus. The total IgG level analysis does not exclude the possibility of an impaired immune response to specific viral infections, particularly when selecting patients with autoimmune diseases. Therefore, in future studies, it would be useful to investigate the presence of the specific antigen/virus and genome detection/sequencing. Additional investigations are required, with larger cohorts of patients/subjects, to clarify whether MWPyV is associated with lymphoproliferative disorders.

## Data availability statement

The raw data supporting the conclusions of this article will be made available by the authors, without undue reservation.

## Ethics statement

This study was carried out in accordance with the recommendations of the County Ethics Committee of Ferrara with written informed consent from all subjects/patients. All subjects/patients gave written informed consent in accordance with the Declaration of Helsinki. The protocol was approved by the County Ethics Committee of Ferrara.

## Author contributions

JN: Investigation, Writing – original draft. EM: Conceptualization, Investigation, Writing – original draft. MI: Investigation, Writing – original draft. RDP: Investigation, Writing – original draft. PG: Investigation, Writing – original draft. NM: Resources, Writing – original draft. CC: Resources, Writing – original draft. EDM: Resources, Writing – original draft. CM: Investigation, Writing – original draft. PC: Conceptualization, Writing – original draft. AT: Conceptualization, Writing – original draft. VB: Investigation, Writing – original draft. MC: Resources, Writing – original draft. MT: Writing – original draft, Conceptualization. FM: Writing – original draft, Conceptualization.
